# The chromatin remodeler Chd1 regulates cohesin in budding yeast and humans

**DOI:** 10.1038/s41598-019-45263-3

**Published:** 2019-06-20

**Authors:** Alexandra Boginya, Rajesh Detroja, Avi Matityahu, Milana Frenkel-Morgenstern, Itay Onn

**Affiliations:** 1 Chromosome Instability and Dynamics Lab. The Azrieli Faculty of Medicine, Bar-Ilan University, Safed, Israel; 2 Cancer Genomics and Biocomputing of Complex Diseases Lab. The Azrieli Faculty of Medicine, Bar-Ilan University, Safed, Israel

**Keywords:** Cohesion, Chromosome condensation, Chromosome segregation, Genomic instability, Chromatin remodelling

## Abstract

Chd1 is a chromatin remodeler that is involved in nucleosome positioning and transcription. Deletion of CHD1 is a frequent event in prostate cancer. The Structural Maintenance of Chromosome (SMC) complex cohesin mediates long-range chromatin interactions and is involved in maintaining genome stability. We provide new evidence that Chd1 is a regulator of cohesin. In the yeast *S. cerevisiae*, Chd1 is not essential for viability. We show that deletion of the gene leads to a defect in sister chromatid cohesion and in chromosome morphology. Chl1 is a non-essential DNA helicase that has been shown to regulate cohesin loading. Surprisingly, co-deletion of CHD1 and CHL1 results in an additive cohesion defect but partial suppression of the chromosome structure phenotype. We found that the cohesin regulator Pds5 is overexpressed when Chd1 and Chl1 are deleted. However, Pds5 expression is reduced to wild type levels when both genes are deleted. Finally, we show a correlation in the expression of CHD1 and cohesin genes in prostate cancer patients. Furthermore, we show that overexpression of cohesin subunits is correlated with the aggressiveness of the tumor. The biological roles of the interplay between Chd1, Chl1 and SMCs are discussed.

## Introduction

**Figure 1:**
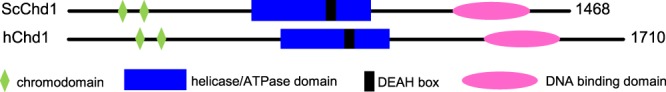
Yeast and human Chd1. Domains are indicated. Sc- *S. cerevisiae*, h- human.

The evolutionary conserved Chd1 gene encodes for an ATP-dependent chromatin-remodeling factor, which contains a chromodomain, a DNA binding domain and a DEAH helicase domain (Fig. 1)^1–3^. The protein is a component of the SAGA and SLIK multi-subunit histone acetyltransferase complexes, which preferentially acetylate histones H3 and H2B, and deubiquitinate histone H2B. Chd1 stimulates DNA unwrapping from the edge of the nucleosome, which is involved in DNA packaging into chromatin, and regulates transcription. Yeast cells lacking CHD1 harbor chromatin defects associated with irregular nucleosome positioning^4,5^. These cells were associated with disparate effects on growth and lifespan^6–10^. In humans, eight members of the CHD family are associated with a variety of cellular functions^1^. Human CHD1 has been identified as a prostate cancer (PCA) tumor suppressor. Loss of CHD1 promotes PCA aggressiveness and is associated with early recurrence of serum prostate specific antigen, a high Gleason grade, advanced tumor stage, and increased cell proliferation^11–13^. Another member of the CHD family, CHD8 has been associated with autism spectrum disorder^14–17^.

While the function of CHD1 in gene expression regulation has been studied extensively, little attention has been given to the effect of the local changes in chromatin structure mediated by Chd1 on the spatial structure of chromatin. The evolutionary-conserved Structural Maintenance of Chromosome (SMC) protein complexes are central factors in the higher order organization of the chromatin^18–20^. The SMC complex cohesin tethers sister chromatids and ensures their bipolar attachment to the spindle and in turn, their accurate segregation; and plays a role in chromosome condensation, DNA repair and transcription regulation^21^. Cohesin is composed of a hetero-dimer of Smc1 and Smc3, a kleisin subunit- Mcd1/Scc1/Rad21 and a set of regulatory HEAT repeat subunits that include Scc3, Wpl1 and Pds5^19,21^.

Chromatin remodelers and helicases can affect cohesin function by several mechanisms. Cohesin loading requires the Scc2/Scc4 loader, whose function includes maintaining a nucleosome free region at the loading site^22–24^. Therefore, nucleosome eviction and translocation may be a prerequisite for cohesin loading. Indeed, the FANCJ-like DNA helicase Chl1/DDX11 has been found to be involved in efficient recruitment of the Scc2/Scc4 loader to the chromatin. It has been suggested that Chl1 affects cohesin loading by chromatin structure in the loading region^25–29^. After loading, cohesin is driven from its loading site by the transcription machinery^30–32^. However, the effects of barriers such as DNA secondary structures, histones and chromatin-bound proteins on cohesin translocation are not fully understood. Chromatin remodelers may be essential for removing such barriers and clear the way for cohesin translocation. Chd1 and Chl1 may be involved in these processes. Indeed, a genetic interaction between Chd1 and cohesin has been reported in several high-throughput screens^33–36^. A physical interaction between Chl1 and cohesin has been found during the S phase of the cell cycle^37^. However, the mechanism by which Chd1 affects cohesin has not been studied.

In this study we identified Chd1 as a new regulator of cohesin in budding yeast and humans. Our findings shed new light on the complexity of cohesin-chromatin complexes and imply a novel mechanism by which a deletion of Chd1 in the prostate drives tumorigenesis through misregulation of cohesin.

## Results

### Deletion of CHD1 induces precocious sister chromatid separation

The interplay between nucleosomes and cohesin motivated us to look for remodelers that may affect SMC activities. Chd1 has been identified as an effector of chromosome stability and a genetic interactor of cohesin in large-scale surveys^34,38,39^. We therefore sought to explore the possibility that Chd1 disturbs the higher order chromatin structure through the activities of the SMC complex cohesin. For this purpose, we constructed a yeast strain in which the CHD1 gene is deleted.

**Figure 2:**
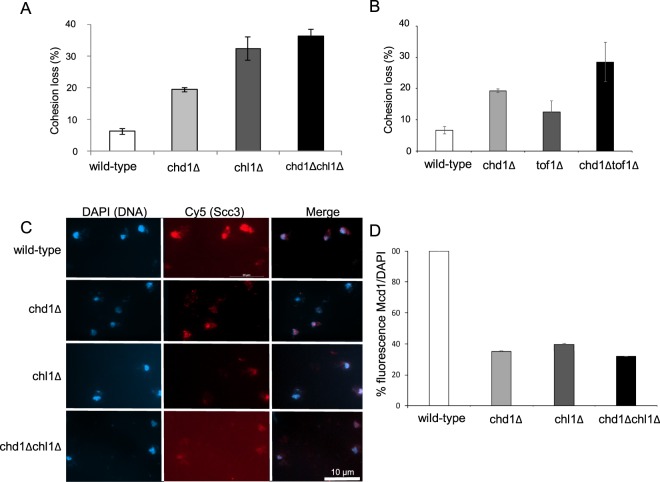
Chd1 and Chl1 deletions affect cohesin activities. (**A**) Strains yIO-120 (wild-type), yAB-2010 (chd1Δ), yAB-2028 (chl1Δ) and yAB-2029 (chd1Δ chl1Δ) were grown to mid-log phase, arrested at the G2/M phase of the cell cycle and processed for cohesion assay (n = 3, P < 0.01). (**B**) Strains, yAM-895 (tof1Δ) and yAM-903(chd1Δ tof1Δ) were grown to mid-log phase, arrested at the G2/M phase of the cell cycle and processed for cohesion assay (n = 3, P < 0.01). (**C**) Strains yIO230 (Scc3-6HA), yIO-2046 (chd1Δ Scc3-6HA**)**, yAB-2045 (chl1Δ Scc3-6HA) and yAB-2048 (chd1Δchl1Δ Scc3-6HA) were grown and arrested at the G2/M phase as in A. Chromosome spreads were prepared and stained with DAPI (DNA) and anti-HA (Scc3); (n = 3, P < 0.01). (**D**) Quantitation of anti-HA (Mcd1) from B. The intensity of Mcd1 was normalized by the intensity of DAPI.

To examine the effect of CHD1 deletion on sister chromatid cohesion we used the GFP dot assay. In brief, an array of LacO was integrated into the LYS4 locus, about 40 kbp distal to the centromere of chromosome III. Wild-type (yIO-120) and chd1Δ (yAB-2010) cells expressing LacI-GFP were used to assess cohesion of cells arrested in G2/M by nocodazole. Normal cohesion is detected as a single GFP dot, while cohesion loss appears as two distinct fluorescent dots. Precocious sister chromatid separation was detected in ~20% of chd1Δ cells, indicating a mild cohesion defect in these cells (Fig. 2A). These levels of cohesion loss are characteristic of deletions of other non-essential cohesion regulators^40^.

The Chl1 helicase has been shown to regulate cohesin, and deletion of this gene results in a similar mild cohesion loss^25–29^. Therefore, we sought to compare the cohesion loss in chd1Δ cells to the effect of CHL1 deletion. For this purpose, we constructed strains yAB-2028 (chl1Δ) and yAB-2029 (chl1Δ chl1Δ), and used them to assess cohesion, as described above (Fig. 2A). In chl1Δ cells, cohesion loss was about 30%. About 36% of the double mutant cells had a cohesion defect.

Genetic interaction between CHD1 and CHL1 can affect cohesion additively or synergistically. The increased loss of cohesion in CHD1Δ CHL1Δ is small compared with the single deletion and therefore, does not fully support an additive effect. To further investigate the effect of CHD1 on this cohesin regulatory pathway, we explored the effect on TOF1, a subunit of the replication-pausing checkpoint complex that also includes Mrc1 and Csm3. TOF1 and CHL1 regulate cohesin in the pathway^40^. We constructed a strain in which TOF1 was deleted alone, or together with CHD1. Cohesion loss was detected in about 13% of tof1Δ cells and in about 30% of chd1Δ tof1Δ cells. These results suggest that CHD1 affects cohesin through the CHL1-TOF1 regulatory pathway.

### Cohesin levels on the chromatin are reduced in CHD1 and CHL1 deleted cells

Chd1 and Chl1 affect nucleosome occupancy and may thus affect cohesin residency on the chromatin. We tagged the Scc3 subunit of cohesin with six hemagglutinin epitopes (HA) in wild type, chd1Δ, chl1Δ and chd1Δchl1Δ cells. Strains yIO-230 (Scc3-HA), yAB-2045 (Scc3-HA chl1Δ), yAB-2046 (Scc3-HA chd1Δ) and yAB-2048 (Scc3-HA chd1Δchl1Δ) were grown to mid-log phase and arrested in G2/M by nocodazole; mitotic chromatin spreads were prepared, stained with anti-HA antibodies and the intensity of cohesin was measured and normalized (Fig. 2C,D). Cohesin loading was affected and reduced by ~60% in chd1Δ and chl1Δ. Similar reduction was observed in chd1Δchl1Δ cells. Based on the cohesion loss and immunostaining results, we concluded that Chd1 is a new effector of cohesin loading.

### CHD1 affects the morphology of the rDNA locus

**Figure 3:**
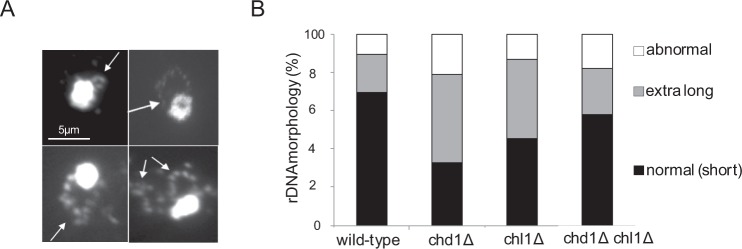
Deletion of CHD1 induces extra-long morphology of the rDNA. Strains yIO-001 (wild-type), yAB-2003 (chd1Δ), yAB-2018 (chl1Δ) and yAB-2019 (chd1Δ chl1Δ) were grown to mid-log phase, arrested at the G2/M phase of the cell cycle, processed for chromosome spreads and stained with DAPI.

In addition to its role in sister chromatid cohesion, cohesin mediates condensation in yeast cells^41–43^. In yeast, the rDNA is located in a single locus on chromosome 12. In mitotic cells, this region appears as a distinguished loop that emerges from the chromosome mass (Fig. 3A). The morphology of the rDNA is determined by genetic and environmental conditions and is used as a marker for mitotic chromosome condensation. Therefore, we tested if deletion of Chd1 and Chl1 also affect condensation.

Strains yIO-001 (wild-type), yAB-2003 (chd1Δ), yAB-2018 (chl1Δ) and yAB-2019 (chd1Δ chl1Δ) were grown at 30 °C and arrested in G2/M by nocodazole. Cells were processed for chromosome spreads and stained with DAPI. We examined the morphology of the rDNA and classified them into three distinct groups: normal (small) loop, extended loop and abnormal loop (Fig. 3A). In wild type cells ~70% of the rDNA revealed the classical short loop structure, ~20% of the rDNA loops were extended and ~10% of the rDNA loops were abnormal, indicating they were decondensed (Fig. 3B). In contrast, spreads from chd1Δ cells revealed classical short loops in only 30% of nuclei; about 50% were extended loops and 21% of the loops were abnormal. A similar percentage, 41%, of extra-long loop morphologies was observed in chl1Δ and chd1Δ nuclei. However, only 13% of the rDNA were abnormal, similar to spreads from wild type cells. Surprisingly, combined deletion of CHD1 and CHL1 partially suppressed the extra-long loop phenotype that was observed in 24% of the nuclei, and the percentage of normal loop morphology was ~60% (Fig. 3B). These results suggest that Chd1 and Chl1 regulate the size of the rDNA loop and have opposing effects on its morphology.

### Deletion of CHD1 and CHL1 affect the DNA damage response but not mitotic recombination

**Figure 4:**
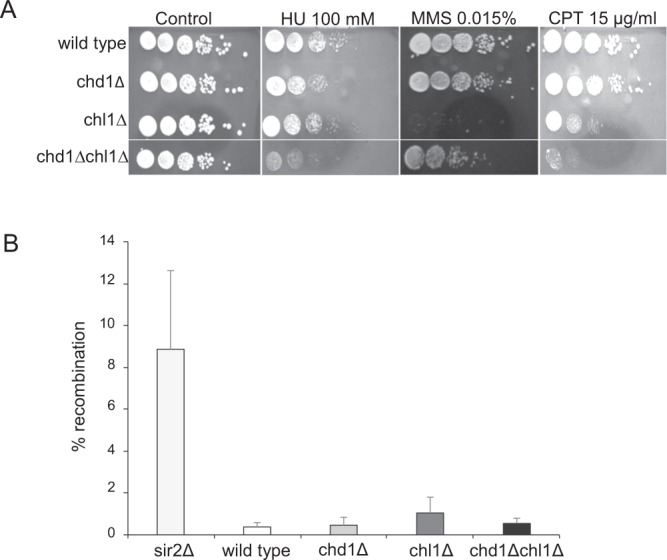
CHD1 and CHL1 are involved in the cell response to genotoxic reagents but not in intra-chromosomal recombination. (**A**) Strains yIO-001 (wild-type), yAB-2003 (chd1Δ), yAB-2018 (chl1Δ) and yAB-2019 (chd1Δ chl1Δ) were grown to saturation, serially diluted and plated on YPD plates with or without the indicated genotoxic drugs. (**B**) Strains yIO-163 (RDN1::ADE2), yIO-164 (sir2∆ RDN1::ADE2), yAB-2039 (chd1∆ RDN1::ADE2), yAB-2044 (chl1∆ RDN1::ADE2) and yAB-2043 (chd1∆ chl1∆ RDN1::ADE2) were grown to saturation and plated on YPD plates. The frequency of intra-chromosomal recombination was measured by the percentage of white/red (ADE2/ade2) cells (n = 3).

Cohesin mutants are sensitive to several DNA damaging reagents. Therefore, we explored the sensitivity of cells carrying a deletion of Chd1 or Chl1 to the genotoxic drugs hydroxyurea (HU), methyl methanesulfonate (MMS) and camptothecin (CPT). These drugs affect the DNA damage response by various mechanisms. HU leads to nucleotide depletion-dependent collapse of the replication fork. MMS induces DNA breaks that activate the S phase checkpoint, while CPT induces DNA breaks that activate the G2 phase checkpoint (Fig. 4A). All single-deletion strains were insensitive to HU, while the chd1Δ chl1Δ double mutant was about tenfold more sensitive than wild type cells. Cells carrying CHD1 deletion were insensitive to MMS and CPT. In contrast, chl1Δ cells were sensitive to these drugs. Interestingly, the double mutant showed a complex phenotype of sensitivity to CPT and HU, and resistance to MMS. We concluded that the deletion of both chd1Δ and chl1Δ cells suppressed the hypersensitivity of chl1Δ cells to S phase DNA breaks induced by MMS. These results suggest a complex genetic interaction between CHD1 and CHL1 in response to DNA damage.

The extended rDNA morphology and alternating condensin levels on the rDNA may affect the frequency of intra-chromosomal recombination between rDNA repeats. To examine this possibility, we inserted the ADE1 gene into the RDN1 locus at chromosome XII. Intra-chromosomal recombination between rDNA copies results in looping out of the ADE1 gene; cells consequently develop a red color^44,45^. We used a sir2Δ strain as a positive control in which the intra-chromosomal recombination frequency is increased compared to the wild type (Fig. 4B). No significant change in recombination frequency was detected in chd1Δ, chl1Δ or chl1Δchl1Δ cells, suggesting that the extended rDNA morphology is not correlated with hyper-recombination in the rDNA.

### Deletion of CHD1 and CHL1 affects PDS5 expression

**Figure 5:**
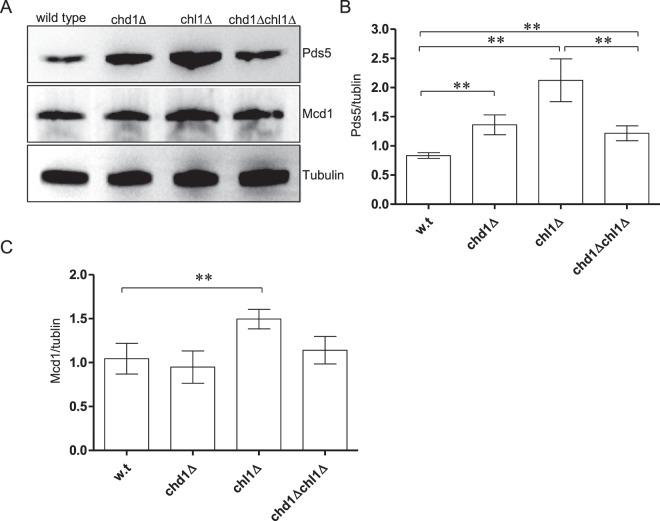
Deletion of CHD1 and CHL1 affects PDS5 expression. (**A**) Strains yOG-3006 (wild-type), yAB-2014 (chd1Δ), yAB-2023 (chl1Δ) and yAB-2024 (chd1Δ chl1Δ) were grown to the mid-log phase, lysed. Equal volumes of extract were loaded and analyzed by SDS-PAGE followed by Western blot against Pds5, Mcd1 and Tubulin. The relative signal of Pds5 (**B**) and Mcd1 (**C**) was detected by using ImageJ (n = 3, P < 0.05).

To dissect the mechanism by which Chd1 and Chl1 affect cohesin, we analyzed protein expression in strains yOG-3006 (CHD1 CHL1), yAB-2014 (chd1Δ), yAB-2023 (chl1Δ) and yAB-2024 (chd1Δ chl1Δ), in cells at mid-log phase. The expression of Mcd1 was not significantly different between wild type and chd1Δ cells. However, Pds5 expression was increased in CHD1Δ and CHL1Δ cells. Interestingly, Pds5 levels were reduced back to the wild type level when both CHD1 and CHL1 were deleted (Fig. 5). These results suggest that Chd1 and Chl1 affect cohesin indirectly by altering the complex stoichiometry.

### Expression of cohesin subunits is dysregulated in PCA

Chd1 has been identified as a PCA tumor suppressor, and loss of CHD1 promotes PCA aggressiveness. We explored a cohort of 502 PCA patients of whom ~23% had a deletion of CHD1. Among the CHD1 deletion samples, 55% were shallow deletions, while the remaining 45% were deep deletions. Deep deletions represented 10% of the total cohort (Sup. Fig. S1).

First, we tested if the expression of genes encoding for cohesin are misregulated in PCA. Expression levels of RAD21 and PDS5B were misregulated in about 10% of the samples, while other cohesin encoding genes were misregulated in 4–9% of the samples. Next, we calculated the correlation between CHD1 deletion and SMC misregulation. In agreement with our finding that CHD1 affects cohesin, we found strong positive correlations of the expression of CHD1 with human cohesin subunits SMC1A, SMC3, STAG1, STAG2, PDS5A, PSD5B and WAPL. The correlation with RAD21 was close to significance (Table 1 and Supplementary Fig. S2). When we analyzed the co-expression of these genes in patients carrying CHD1 deep deletion, this correlation remained significant (Table 1 and Supplementary Fig. S2). Interestingly, we did not find a correlation between the expression of CHD1 and CHL1/DDX11. As a control, we calculated correlations between CHD1 expression and condensin subunits. No significant correlation was found in these analyses (Table 1 and Supplementary Fig. S3). These results suggest that the regulation of cohesin through CHD1 is conserved from yeast to humans.

**Table 1:** Correlation between expression of CHD1, cohesin and condensin encoding genes in PCA samples.

Gene*	PCA samples (total)	*P*-value	PCA samples with CHD1Δ	*P*-value
**CHL1/DDX11**				
DDX11	0.05	0.216	−0.16	0.2469
**Cohesin core subunits**				
*SMC1A*	*0.7*	*2.2e-16*	*0.5*	*0.0002088*
*SMC3*	*0.67*	*2.2e-16*	*0.52*	*8.849e-05*
RAD21**	0.41	2.2e-16	0.47	0.000465
*STAG1*	*0.73*	*2.2e-16*	*0.51*	*0.0001186*
*STAG2*	*0.67*	*2.2e-16*	*0.59*	*6.446e-06*
**Cohesin regulators**				
*WAPL*	*0.68*	*2.2e-16*	*0.63*	*8.289e-07*
*PDS5A*	*0.75*	*2.2e-16*	*0.57*	*1.133e-05*
*PDS5B*	*0.57*	*2.2e-16*	*0.61*	*2.469e-06*
*NIPBL*	*0.61*	*2.81e-53*	*0.55*	*0.0007*
*ESCO1*	*0.42*	*2.61e-22*	*0.48*	*0.004*
**Condensin**				
SMC2	0.44	2.2e-16	0.44	0.001371
SMC4	0.42	2.2e-16	0.34	0.01378
**Condensin I**				
NCAPH	0.06	0.1251	−0.13	0.3626
NCAPD2	0.42	2.2e-16	0.04	0.0033
NCAPG	0.12	0.006	0.02	0.864
**Condensin II**				
NCAPH2	−0.36	2.2e-16	−0.31	0.02793
NCAPD3	−0.02	0.62	0	0.945
NCAPG2	0.36	2.2e-16	0.21	0.1401

*Genes with significant Pearson’s correlations are in *Italic*.

**Pearson’s correlation is close to statistical significance of 0.5.

To gain a comprehensive view of the interplay between CHD1 and cohesin in PCA, the expression correlation analysis was extended to cohesin auxiliary factors. NIPBL and MAU2 mediate cohesin loading. We found a strong correlation between expression levels of CHD1 and NIPBL. Weak correlation was found with MAU2 in PCA samples but not in CHD1Δ samples (Table 1 and Supplementary Fig. S2). The acetyltransferases ESCO1 and ESCO2 regulate cohesin activity. While both ESCO1 and ESCO2 are important for sister chromatid cohesion, ESCO1 is essential for non-mitotic cohesin activities such as DNA repair and gene expression regulation^46^. Interestingly, CHD1 expression was found to be correlated with ESCO1, but not with ESCO2, in PCA patients (Table 1 and Supplementary Fig. S2).

Finally, we examined the effect of cohesin subunit expression level on the survival of PCA patients. No significant difference was detected in disease/progression-free Kaplan-Meier estimates (data not shown). However, a significant effect on tumor aggressiveness, as indicated by a high Gleason score, was found in low cohesin expressing PCA samples (Supplementary Fig. S4). These results suggest that deletion of CHD1 results in reduced expression of cohesin, and a subsequent increase in tumor aggressiveness.

### PDS5B deletion is common in PCA

We found a correlation between expression levels of CHD1 and PDS5 in PCA patients. This prompted us to explore the effects of PDS5 deletion on PCA. CHD1 was deleted in 6.56% of the patients. The frequency of PDS5A deep deletion was 0.4%, while PDS5B was deleted in 3.89% of the samples. These results suggest that PDS5B deletion is a newly identified driver event in PCA development.

Next, we explored the deletion frequency of the examined genes in all cancer types (32 TCGA PanCancer Atlas Studies). CHD1 deletion was most common in PCA. However, the frequencies were lower in 50% of the different cancer types. Similar to PCA, PDS5A deletion was detected in lower frequencies in 20% of cancer types. Similar to CHD1, PDS5B deletion was detected in 50% of the cancer types; the highest deletion frequencies were in PCA, sarcoma and diffuse large B-cell lymphoma. These results suggest that PDS5B is a newly identified driver of cancer with an important role in PCA.

### CHD1 and PDS5B are connected through PCNA

**Figure 6:**
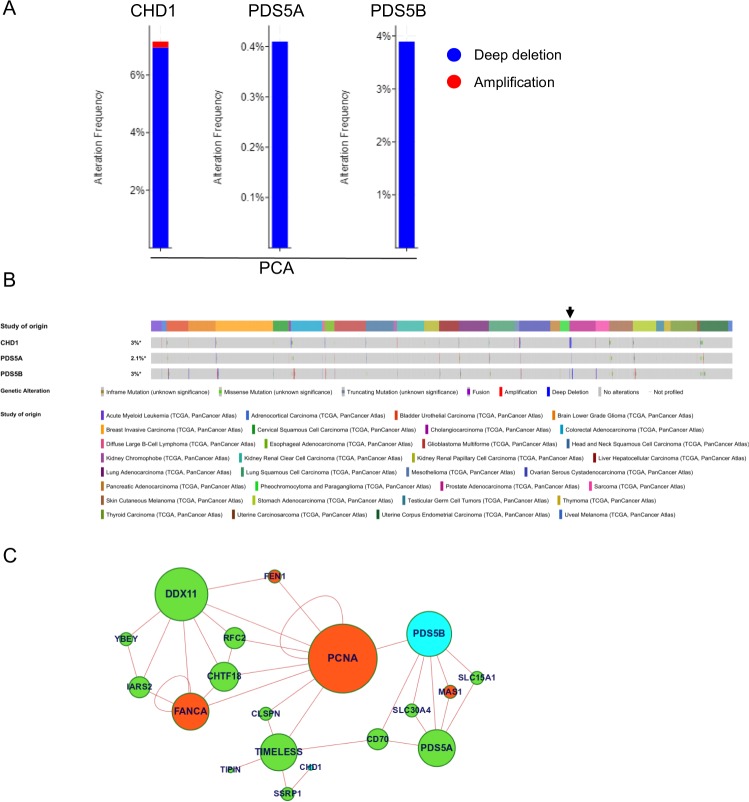
Deletions of CHD1 and PDS5B are common in PCA patents. (**A**) Frequencies of copy number alternations of CHD1, PDS5A and PDS5B were tested in 494 samples of PCA patients. (**B**) Copy number alternations of these genes were tested in 32 TCGA PanCancer Atlas studies containing 10,528 samples. (**C**) Protein-protein interaction network analysis shows the cellular connection between CHD1 and PDDS5, mediated by the Chimeric Protein-Protein Interaction method (ChiPPI). The full network is shown in Supplementary Fig. S7.

To better understand the interplay between PDS5 and CHD1, we performed a network analysis. We used the Chimeric Protein-Protein Interaction method (ChiPPI)^47^ to predict alterations to the cellular networks. We considered the cohesin complex and the CHD1 deletion that is found in many cancers, and that is particularly frequent in PCA. According to this approach, pre-computed data from multiple PubMed references on tumor-suppressors and oncogenes are used to identify gene roles in cancer. Based on this analysis, we found that CHD1 and PDS5 are connected through TIMLESS (the human homolog of TOF1) and the DNA polymerase loader clamp PCNA. Furthermore, the analysis indicated that the CHD1 is an oncogene and that PDS5B is a tumor suppressor (Fig. 6C and Supplementary Fig. S7). Altogether, these results imply that CHD1 and PDS5 cooperate in cancer development.

## Discussion

We identified the chromatin remodeler Chd1 as a new regulator of the SMC complex cohesin in yeast. We showed that deletion of CHD1 affects cohesin chromosome binding, similar to the effect of the known cohesin regulator Chl1. Furthermore, double deletion of CHD1 and either CHL1 or TOF1 affected precocious chromatid separation. These results suggest that in yeast, CHD1 affects cohesin through the CHL1-TOF1-CSM3-CTF4. The bioinformatics analysis indicates that this effect is conserved in humans. Deletion of CHD1 also affects the morphology of the rDNA locus. Surprisingly, co-deletion of CHL1 suppresses this phenotype. Chd1 and Chl1 activity affect nucleosome occupancy and positioning. Therefore, we suggest that local changes in nucleosomes can affect the higher order structure of chromatin by affecting cohesin function.

Chd1 is an essential component of the histone acetyltransferase complexes SAGA and SLIK, which preferentially acetylate histones H3 and H2B, and deubiquitinate histone H2B^2^. Chd1 is involved in chromatin remodeling, nucleosome sliding, gene expression and transcriptional elongation. Deletion of the CHD1 encoding gene is associated with extended heterochromatin regions. A number of reports have claimed that Chd1 prevents exchange of histones in the coding region^4,5,48,49^. Chd1 can affect cohesin by several mechanisms. Chromatin remodeling may affect cohesin loading and translocation. Chl1 has been shown to interfere with Scc2/Scc4 loading, which requires nucleosome free DNA for efficiency^23,29^. We cannot rule out the possibility that Chd1 has a similar effect. Another mechanism may be associated with cohesin translocation from its loading sites^31,32^. Evidence suggests that DNA structures and chromatin bound proteins may block the free movement of cohesin. In such case, irregular nucleosome orientation may act as a barrier. The RSC chromatin remodeler complex has been identified as an important factor for cohesin translocation^5,23^. It is possible that the activity of Chd1, Chl1 and most likely other remodelers may similarly affect cohesin association with chromatin. In agreement with this hypothesis, components of the RSC complex interact genetically with Chd1, implying redundancy between remodelers.

In addition to the possible interplay between chromatin structure and cohesin activity, Chd1 is a transcription regulator. We noted that deletion of the gene leads to a 1.5 fold increase in levels of Pds5. A similar effect was observed in CHL1 deleted cells. It has been suggested that Pds5 competes with Scc2 on the same cohesin binding site^24,50^. Overexpression of Pds5 may shift complex stoichiometry and enhance cohesin chromatin dissociation over association.

A notable effect of CHD1 deletion is evident by the morphology of the rDNA. The size of the rDNA loop has been shown to be affected by temperature. We demonstrated that genetics can also contribute to the size of the loop. However, cohesin levels per se seem not to sufficiently explain rDNA morphology, as cohesin levels were similarly reduced in the strains in which CHD1, and both CHD1 and CHL1, were deleted. Nonetheless, a difference in the frequency of extended loop morphology was found between these strains. The biological significance of the loop size is yet to be determined. Loop size has been shown to be affected by temperature and may reflect the transcription level of the rDNA genes^51^. We speculated that the extended rDNA structure may be associated with intra-recombination. We detected a mild increase in recombination frequency in chl1Δ cells. However, this change was not statistically significant and thus, this hypothesis was not confirmed. The opposing effect of CHD1 and CHL1 on rDNA morphology, as was found in the double mutant, suggests that these proteins may differently regulate cohesin in this region. Further, genetic studies are needed to determine the genetic factors that regulate the size of the rDNA loop and to understand the biological differences between compact and extended loop morphologies.

One of the interesting findings of this study relates to the interplay between Chd1 and cohesin in PCA. Deletion of the CHD1 gene is a common event in PCA, which is one of the most frequent malignancies in men worldwide. PCA carrying a CHD1 deletion is associated with early recurrence of serum prostate specific antigen, a high Gleason grade, advanced tumor stage and increased cell proliferation^11–13^. However, the molecular mechanism by which the deletion affects the tumor is not fully understood.

Our analysis on PCA samples supports the idea that at least some of these clinical properties are related to cohesin misregulation. We found positive correlations of CHD1 with several cohesin subunits and regulators. particularly, cohesin can affect tumor aggressiveness by inducing genome instability, and also through misregulation of oncogenes and tumor suppressors^52^. The correlations of CHD1 expression with PDS5B and ESCO1, but not with ESCO2, support associations of clinical outcomes in CHD1Δ patients with non-mitotic functions of cohesin. The contribution of cohesin to PCA is in addition to its involvement in leukemia, bladder cancer and other malignancies^53–56^. Remarkably, we found that deletion of the cohesin regulator PDS5B is a common event in PCA, as well as in other types of cancer. This adds to the growing number of reports on the involvement of this gene in cancer^57–60^. The identification of cohesin as a factor in PCA progression is important for the development of biomarkers and personalized medicine approaches for treating this type of cancer.

## Materials and Methods

### Yeast strains, cell growth and synchronization

The yeast strains that were used in this study are listed in Supplementary Table 1. Cells were grown in either liquid or solid YPD. When indicated, the YPD plates were supplemented by 100 mM HU (Bio-Basic), 0.015% methyl methanesulfonate (Sigma) or 15 μg/ml camptothecin (Sigma). For G2/M arrests, cells were grown to the mid-log phase and arrested by supplementing the growth medium with 15 μg/ml nocodazole (Sigma). Genes were deleted by using standard transformation protocol and verified by PCR.

### Sister chromatid cohesion assay

Sister chromatid cohesion was measured by using the GFP dot assay as previously reported^43,61,62^. In brief, cells carrying LacI-GFP and LacO array, integrated at the LYS2 locus, about 40 kb from the centromere, were grown to mid-log phase and arrested at G2/M by 5 µM nocodazole. Cells were fixed by using 4% formaldehyde and washed once with KPO_4_/sorbitol solution (0.1 M KPO_4_ pH = 7.5 and 1.2 M sorbitol), resuspended in KPO_4_/sorbitol and stored at 4 °C. Samples were analyzed using AxioImager M2 microscope (Zeiss), magnification x100, N/A = 1.4.

### Chromosome spreads and immunostaining

Yeast strains were grown in YPD to the mid-log phase and arrested in the G2/M phase of the cell cycle by nocodazole. Chromosome spreads were prepared as described in^43^ and nuclei stained with DAPI. For immunostaining, slides were washed with PBS for 30 min and then blocked with 20% BSA for 10 min at room temperature. The slides were covered with mouse anti-HA antibody (Roche) and incubated in a humid chamber for 1 hr at 23 °C, washed with PBS for 10 min at room temperature, and re-incubated with a goat anti-mouse antibody conjugated to Alexa Fluor 680 (Life Technologies) for 1 hr at 23 °C. Slides were washed with a PBS for 10 min, dried and covered with 100 ng/ml prolong gold antifade reagent DAPI. Nuclei were observed by AxioImager M2 microscope (Zeiss), magnification x100, NA = 1.4.

### Intra-chromosomal recombination

Intra-chromosomal recombination was described in^63,64^. In brief, the ADE2 gene is inserted into the rDNA locus. Cells expressing ADE2 are white. Cells in which the ADE2 gene is lost as a result of intra-molecular recombination develop a red color. Cell cultures were grown to saturation and plated on YPD plates, incubated at 30 °C for 3–4 days, then transferred to 4 °C until the red color appeared. The frequency of intra-chromosomal recombination was measured as the ratio between white (ADE2) and red (ade2) cells.

### Western blot

Protein extracts from mid-log cells were prepared and analyzed by SDS-PAGE followed by Western blot. Polyclonal antibodies against Mcd1 and Pds5 were a generous gift from Vincent Guacci. Antibody against tubulin was purchased from Abcam. Densitometry was done with Image J software. Statistical significance was determined by t-test on the average of 3 independent experiments. Reprehensive full blots are shown in Supplementary Fig. S8.

### Bioinformatics

Putative CHD1 copy-number alterations from GISTIC for prostate adenocarcinoma of the Cancer Genome Atlas (TCGA, Provisional, 499 samples) were calculated using cBioPortal for cancer genomics^65,66^. Fifty (10%) of the patients were retrieved with CHD1 deletion. Further, mRNA expression data for a total of 502 PCA patients of TCGA were downloaded from NCBI’s GEO DataSets (GSE62944). The gene expression of cohesin and condensin subunits for all 502 patients were compared to the that of patients with CHD1 deletion by using in-house R script; the Pearson correlation coefficient was calculated. Pearson’s correlation coefficient in the range of 0.1–0.3 was considered as weak, 0.3–0.5 as moderate and 0.5–1.0 as a strong correlation. Moreover, based on the gene expression values, Kaplan-Meier survival plots were generated for PCA patients using the UALCAN server^67^.

### Network analysis of cohesion interactions in human cells

To calculate protein-protein interactions (PPIs) and their alterations due to gene deletions in cancers, we used our previously developed method, ChiPPI^47^. In brief, ChiPPI utilizes a pre-computed domain-domain co-occurrence table based on all human interactions from BioGrid^68^ and evaluates all the missing and gained interactors of the deleted genes in cellular PPI networks.

## Supplementary information

Supplementary Information
