# Supplementary material for: The chromatin remodeler Chd1 regulates cohesin in budding yeast and humans
Source: Sci Rep. 2019 Jun 20;9:8929. doi: 10.1038/s41598-019-45263-3 (PMC6586844; doi:10.1038/s41598-019-45263-3)
Supplement: Supplementary file 1 — Supplementary Information [file 41598_2019_45263_MOESM1_ESM.pdf]

The chromatin remodeler Chd1 regulates cohesin in budding yeast and humans.

Alexandra Boginya[1], Rajesh Detroja[2], Avi Matityahu[1], Milana Frenkel-Morgenstern[2] and Itay Onn[1*]

1. Chromosome Instability and Dynamics Lab. The Azrieli Faculty of Medicine, Bar-Ilan University, Safed, Israel

2. Cancer Genomics and BioComputing of Complex Diseases lab. The Azrieli Faculty of Medicine, Bar-Ilan University, Safed, Israel

Supplementary information

Figure S1. Copy number alternation in the PCA cohort. 20% of the samples carried either a shallow or deep deletion of CHD1**.**

Figure S2. Correlates of the expression of CHD1, cohesin subunits and auxiliary factors, with prostate cancer (PCA) aggressiveness. For all genes, the left panel shows result**s** in a cohort of 502 PCA tumors, while the right panel shows result**s** in a cohort of 50 PCA tumors in which CHD1 was deleted**.** The gene name is indicated in the graph. The P-value is indicated

Figure S3. Correlates of the expression of CHD1, condensin I and condensin II subunits, with prostate cancer (PCA) aggressiveness. For all genes, the left panel shows the results in a cohort of 502 PCA tumors, while the right panel shows the results in a cohort of 50 PCA tumors in which CHD1 was deleted**.** The gene name is indicated on the graph. The P-value is indicated

Figure S4. Correlates of the expression of cohesin encoding genes with Gleason grade score and survival probability. The Gleason score of the tumor and expression levels of the indicated genes were plotted against survival probability. The gene name is indicated on the graph. The P-value is indicated

Figure S5. Uncropped membranes shown in Fig. 5. A. anti-Pds5. **B.** anti-Mcd1. **B.** anti-tubulin.

Figure S6. Analysis of mutations in CHD1, PDS5A and PDS5B in different cancer types. Mutation types were analyzed in CHD1, PDS5A and PDS5B in 32 TCGA PanCancer Atlas studies, containing 10,528 samples. The mutation frequency for each cancer type is shown.

Figure S7. Protein-protein interaction network analysis. The full protein-protein interaction network analysis mediated by the Chimeric Protein-Protein Interaction method (ChiPPI). The network shown in Fig. 6C was derived from this analysis.

Supplementary Fig. S1

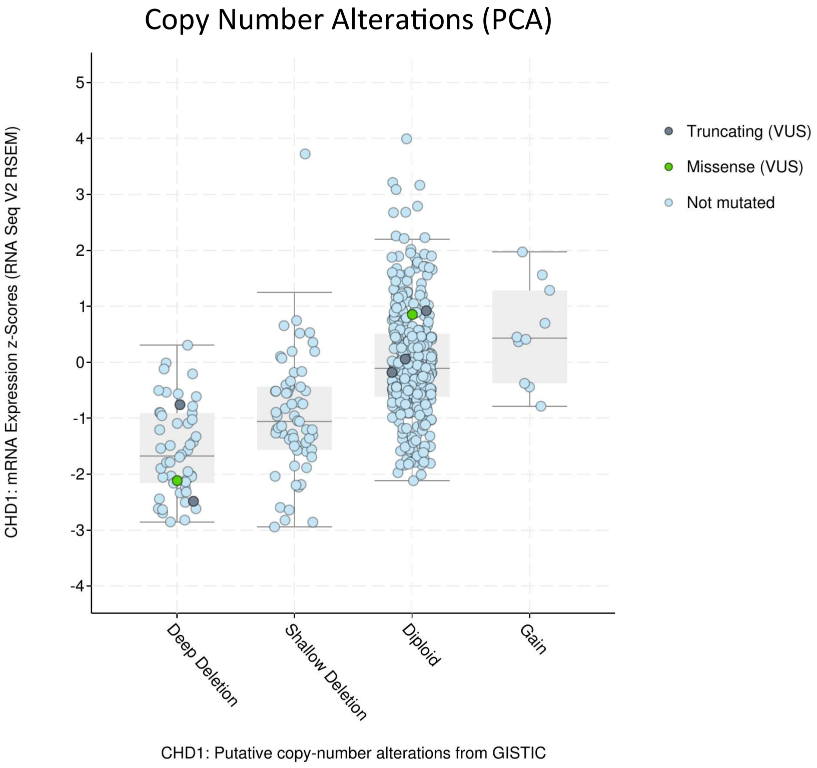

Figure S2

Cohesin core subunits

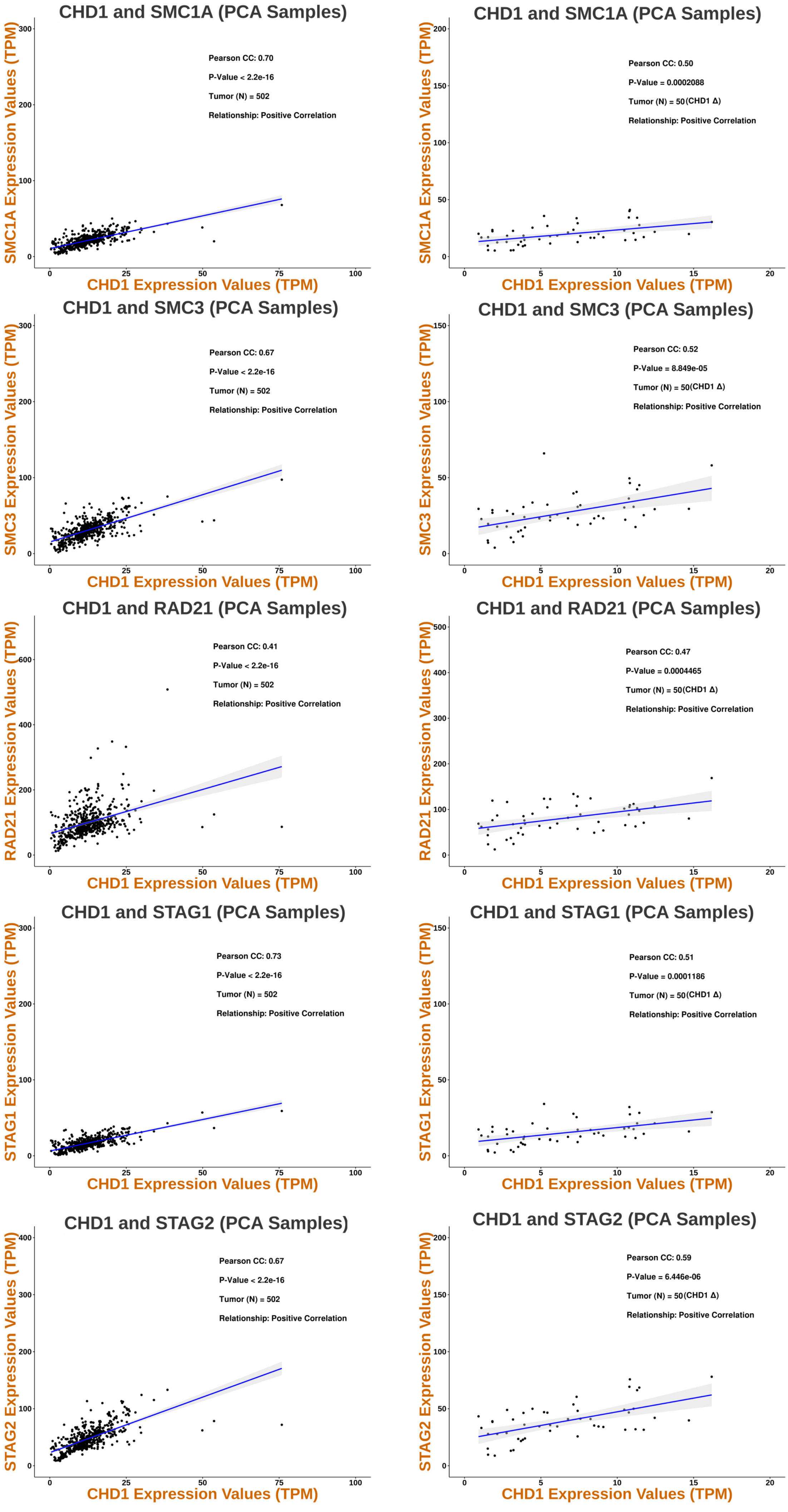

WAPL (cohesin regulatory subunit)

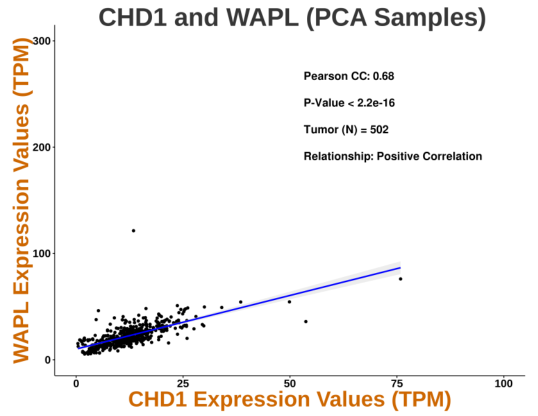

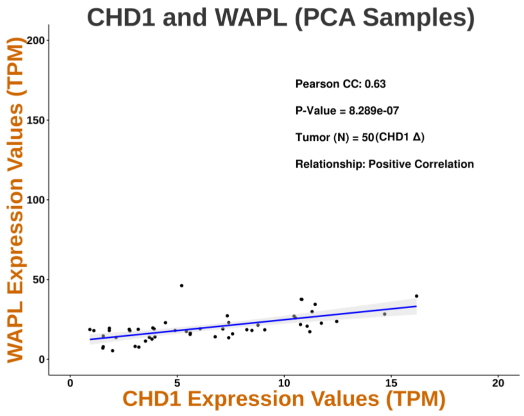

PDS5A/B (cohesin regulatory subunit)

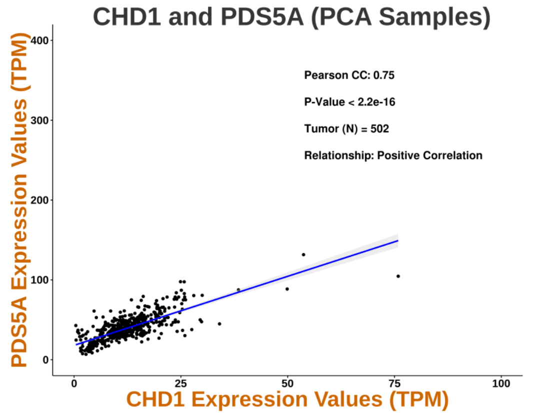

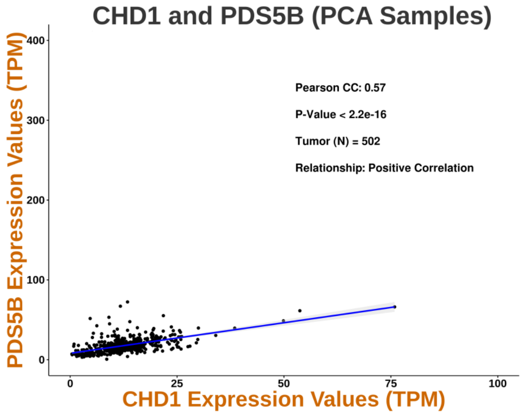

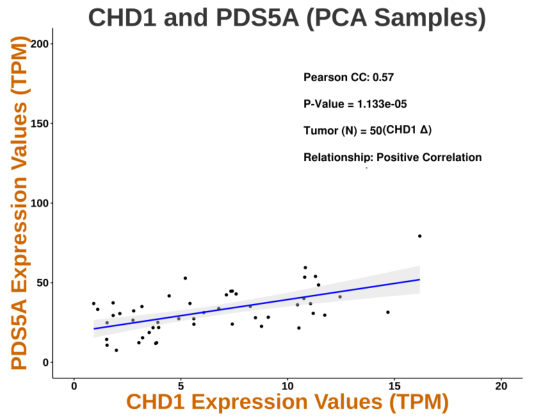

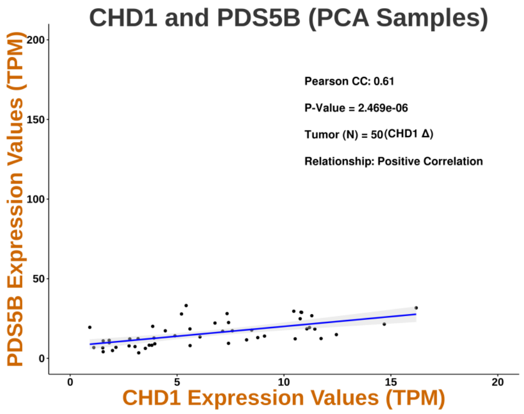

CHL1/DDX11

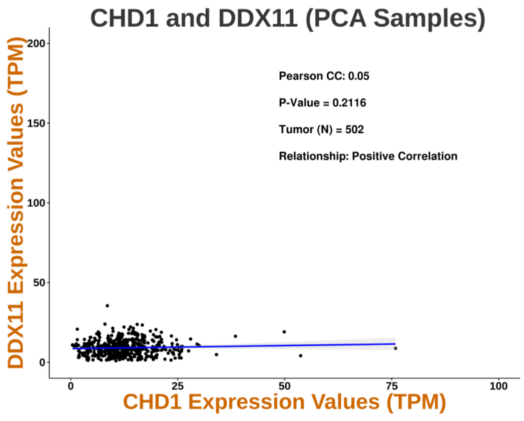

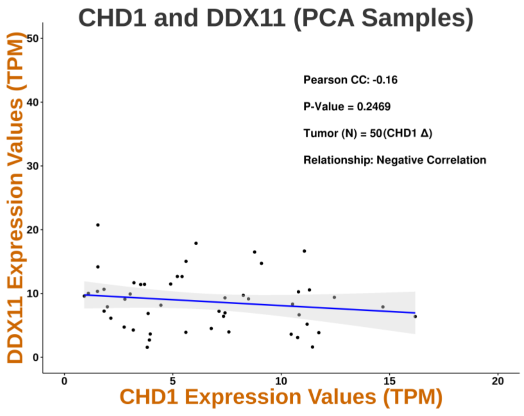

Cohesin Loader

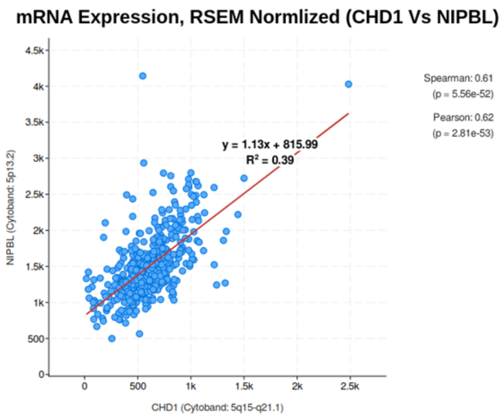

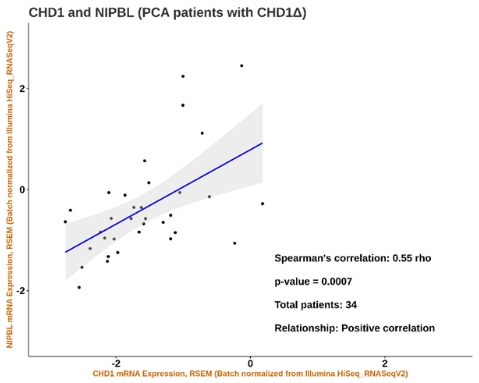

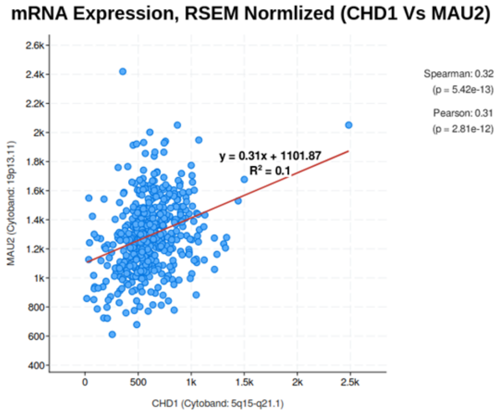

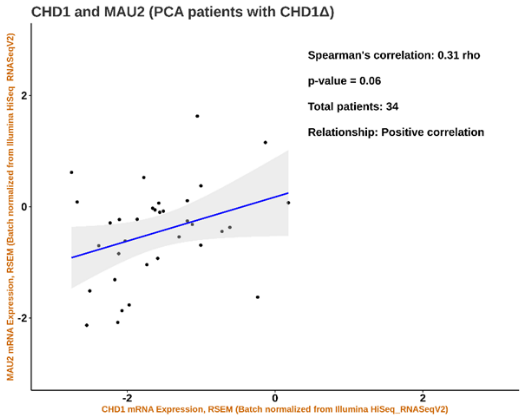

Cohesion Establishment

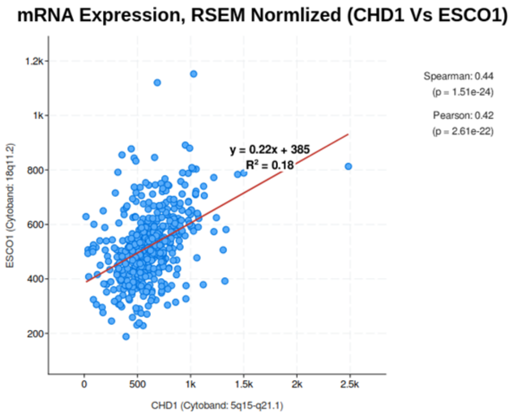

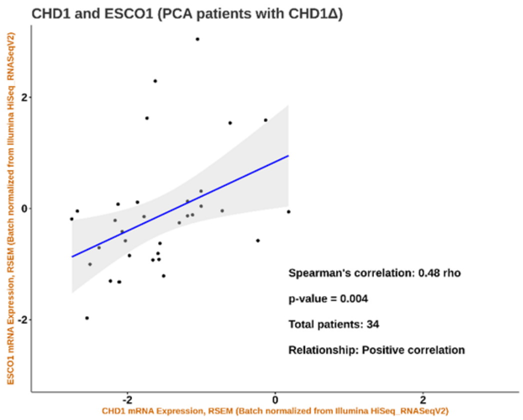

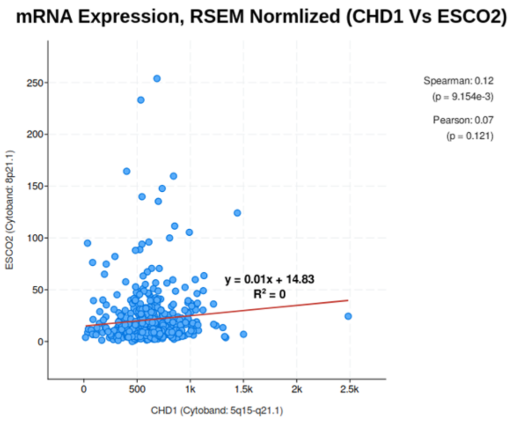

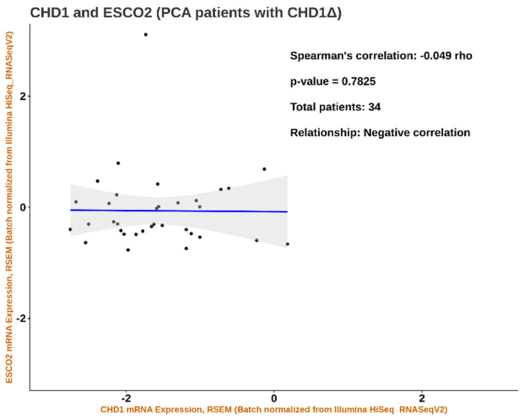

Figure S3

Condensin SMC subunits

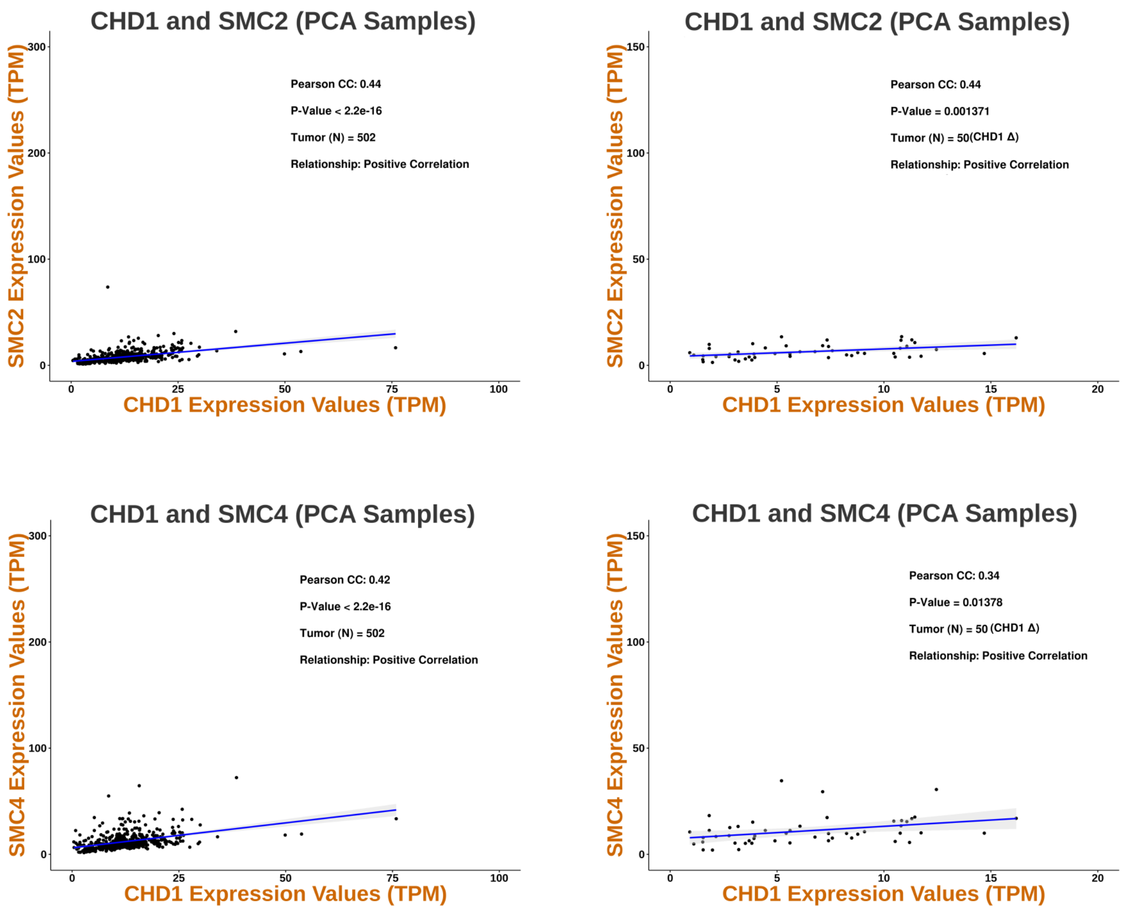

Condensin I subunits

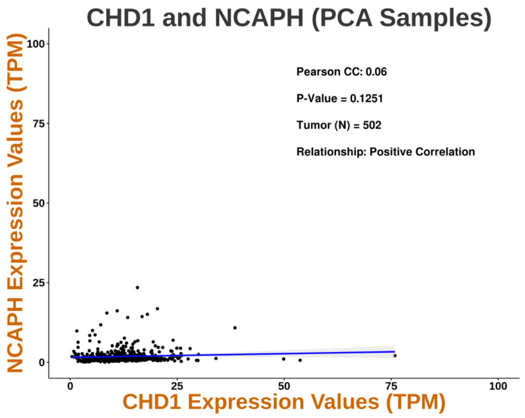

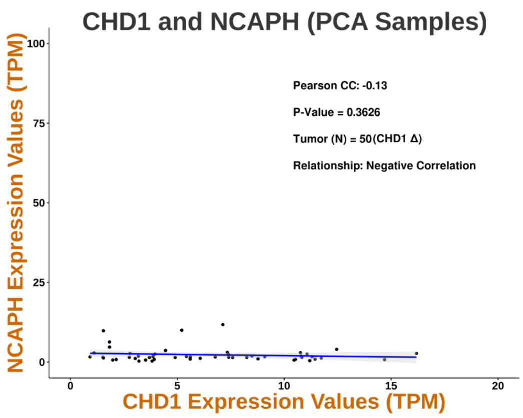

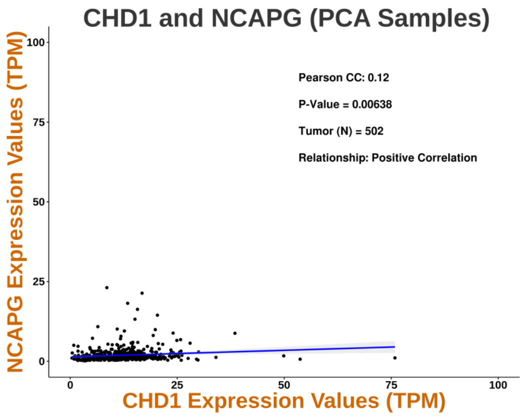

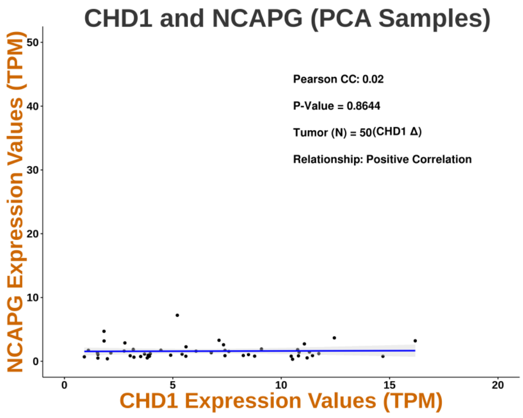

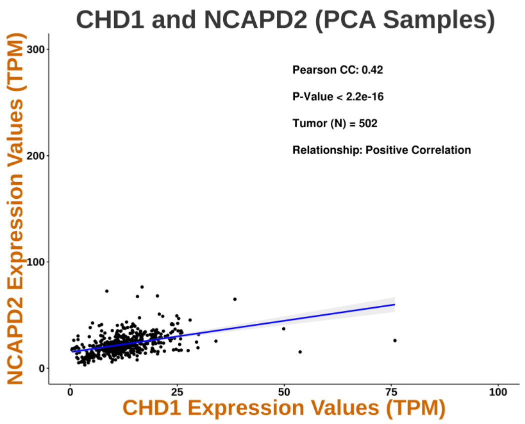

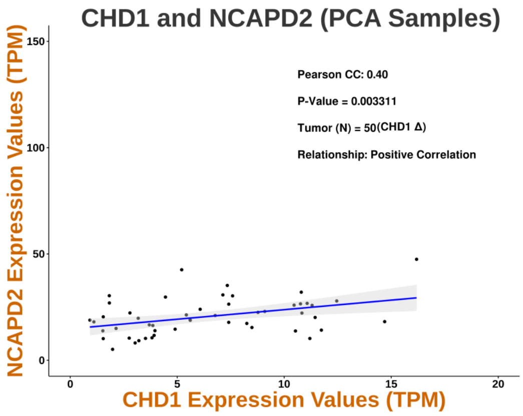

Condensin II subunits

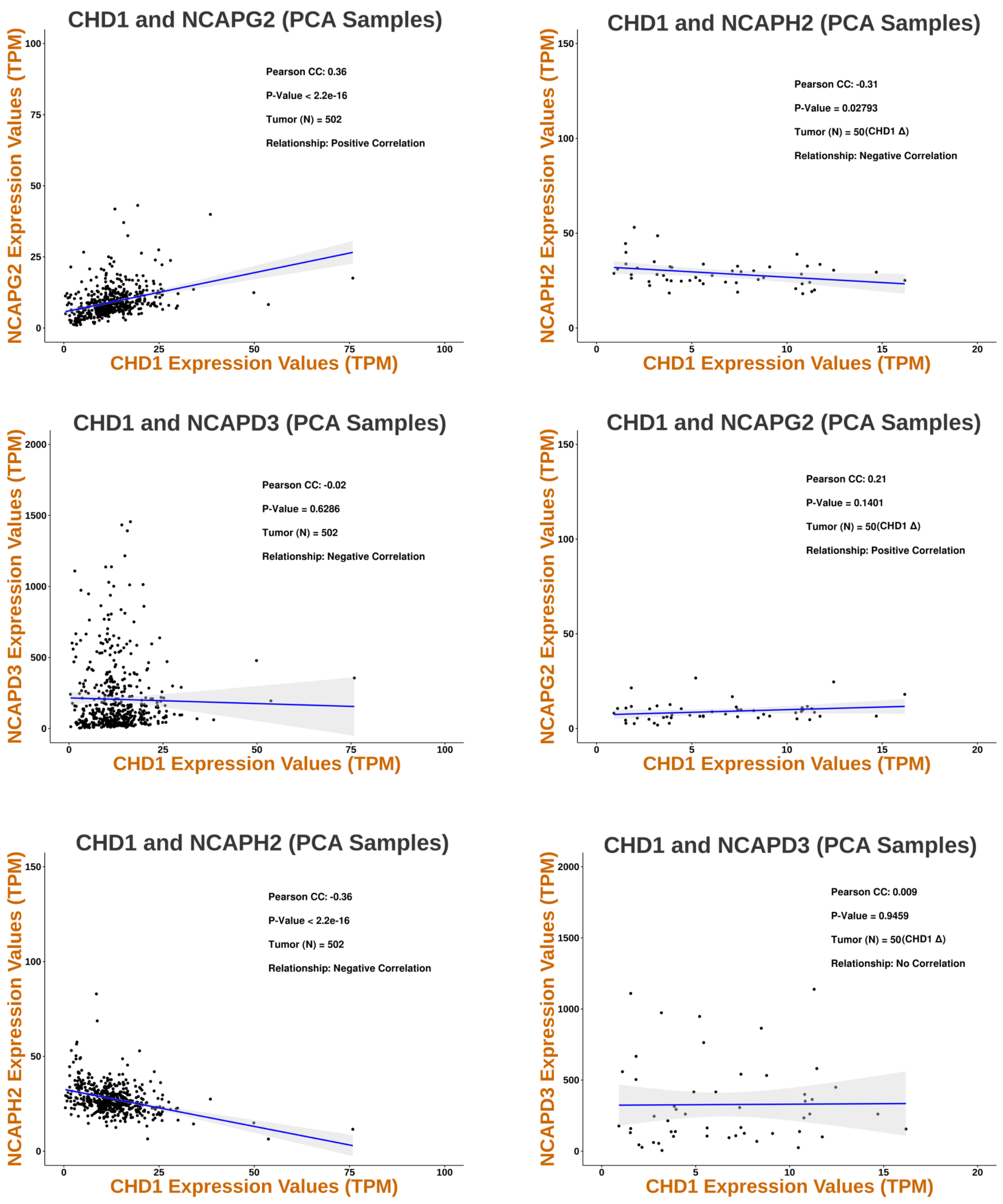

Figure S4

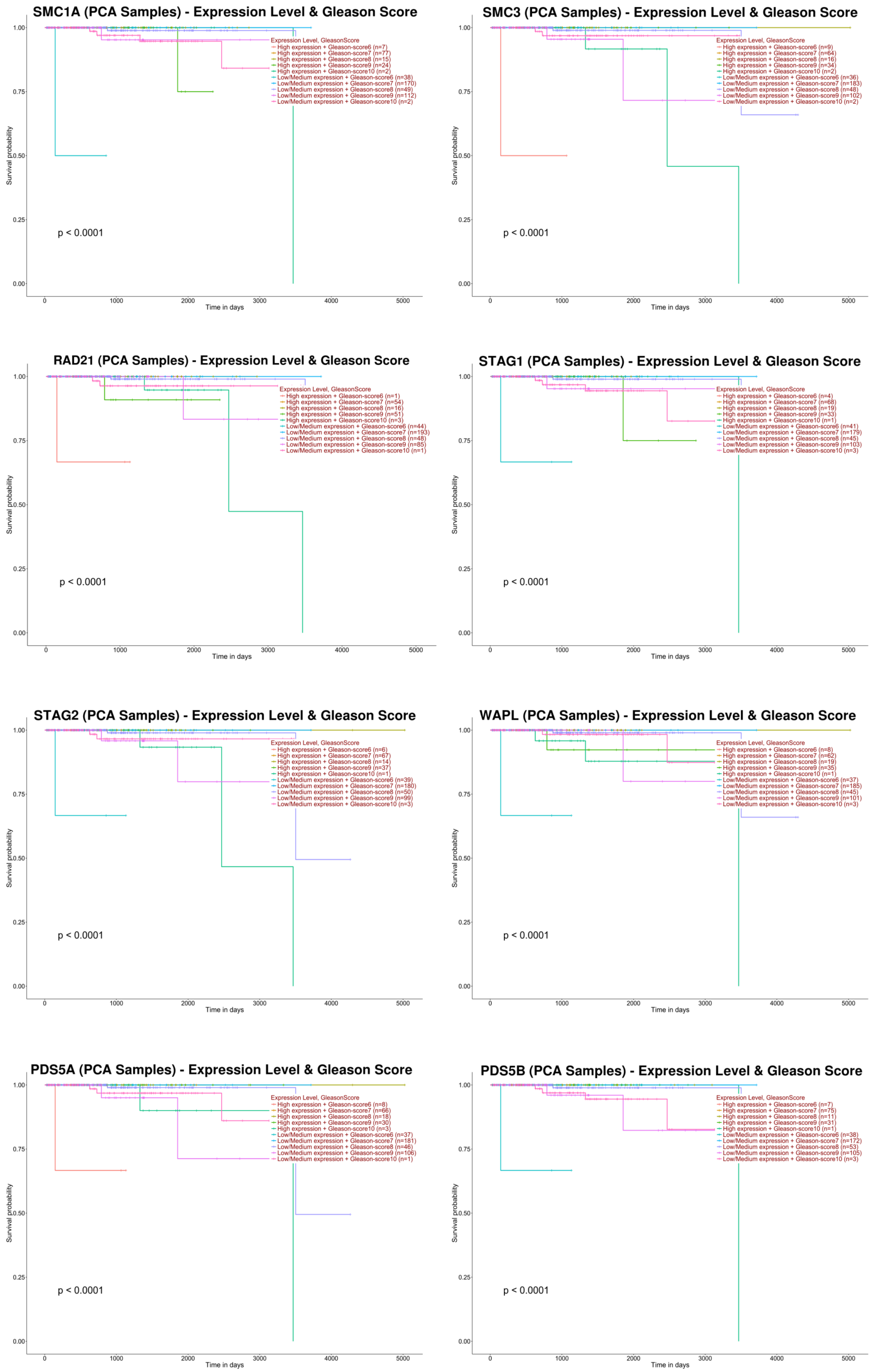
SMC1A (PCA Samples) - Expression Level & Gleason Score
Survival probability
Time in days
p < 0.0001
Expression Level, GleasonScore
High expression + Gleason-score6 (n=7)
High expression + Gleason-score7 (n=77)
High expression + Gleason-score8 (n=15)
High expression + Gleason-score9 (n=24)
High expression + Gleason-score10 (n=2)
Low/Medium expression + Gleason-score6 (n=38)
Low/Medium expression + Gleason-score7 (n=170)
Low/Medium expression + Gleason-score8 (n=49)
Low/Medium expression + Gleason-score9 (n=112)
Low/Medium expression + Gleason-score10 (n=2)
SMC3 (PCA Samples) - Expression Level & Gleason Score
Survival probability
Time in days
p < 0.0001
Expression Level, GleasonScore
High expression + Gleason-score6 (n=9)
High expression + Gleason-score7 (n=64)
High expression + Gleason-score8 (n=16)
High expression + Gleason-score9 (n=34)
High expression + Gleason-score10 (n=2)
Low/Medium expression + Gleason-score6 (n=36)
Low/Medium expression + Gleason-score7 (n=183)
Low/Medium expression + Gleason-score8 (n=48)
Low/Medium expression + Gleason-score9 (n=102)
Low/Medium expression + Gleason-score10 (n=2)
RAD21 (PCA Samples) - Expression Level & Gleason Score
Survival probability
Time in days
p < 0.0001
Expression Level, GleasonScore
High expression + Gleason-score6 (n=1)
High expression + Gleason-score7 (n=54)
High expression + Gleason-score8 (n=16)
High expression + Gleason-score9 (n=51)
High expression + Gleason-score10 (n=3)
Low/Medium expression + Gleason-score6 (n=44)
Low/Medium expression + Gleason-score7 (n=193)
Low/Medium expression + Gleason-score8 (n=48)
Low/Medium expression + Gleason-score9 (n=85)
Low/Medium expression + Gleason-score10 (n=1)
STAG1 (PCA Samples) - Expression Level & Gleason Score
Survival probability
Time in days
p < 0.0001
Expression Level, GleasonScore
High expression + Gleason-score6 (n=4)
High expression + Gleason-score7 (n=68)
High expression + Gleason-score8 (n=19)
High expression + Gleason-score9 (n=33)
High expression + Gleason-score10 (n=1)
Low/Medium expression + Gleason-score6 (n=41)
Low/Medium expression + Gleason-score7 (n=179)
Low/Medium expression + Gleason-score8 (n=45)
Low/Medium expression + Gleason-score9 (n=103)
Low/Medium expression + Gleason-score10 (n=3)
STAG2 (PCA Samples) - Expression Level & Gleason Score
Survival probability
Time in days
p < 0.0001
Expression Level, GleasonScore
High expression + Gleason-score6 (n=6)
High expression + Gleason-score7 (n=67)
High expression + Gleason-score8 (n=14)
High expression + Gleason-score9 (n=37)
High expression + Gleason-score10 (n=1)
Low/Medium expression + Gleason-score6 (n=39)
Low/Medium expression + Gleason-score7 (n=180)
Low/Medium expression + Gleason-score8 (n=50)
Low/Medium expression + Gleason-score9 (n=99)
Low/Medium expression + Gleason-score10 (n=3)
WAPL (PCA Samples) - Expression Level & Gleason Score
Survival probability
Time in days
p < 0.0001
Expression Level, GleasonScore
High expression + Gleason-score6 (n=8)
High expression + Gleason-score7 (n=62)
High expression + Gleason-score8 (n=19)
High expression + Gleason-score9 (n=35)
High expression + Gleason-score10 (n=1)
Low/Medium expression + Gleason-score6 (n=37)
Low/Medium expression + Gleason-score7 (n=185)
Low/Medium expression + Gleason-score8 (n=45)
Low/Medium expression + Gleason-score9 (n=101)
Low/Medium expression + Gleason-score10 (n=3)
PDS5A (PCA Samples) - Expression Level & Gleason Score
Survival probability
Time in days
p < 0.0001
Expression Level, GleasonScore
High expression + Gleason-score6 (n=8)
High expression + Gleason-score7 (n=66)
High expression + Gleason-score8 (n=18)
High expression + Gleason-score9 (n=30)
High expression + Gleason-score10 (n=3)
Low/Medium expression + Gleason-score6 (n=37)
Low/Medium expression + Gleason-score7 (n=181)
Low/Medium expression + Gleason-score8 (n=46)
Low/Medium expression + Gleason-score9 (n=106)
Low/Medium expression + Gleason-score10 (n=1)
PDS5B (PCA Samples) - Expression Level & Gleason Score
Survival probability
Time in days
p < 0.0001
Expression Level, GleasonScore
High expression + Gleason-score6 (n=7)
High expression + Gleason-score7 (n=75)
High expression + Gleason-score8 (n=11)
High expression + Gleason-score9 (n=31)
High expression + Gleason-score10 (n=1)
Low/Medium expression + Gleason-score6 (n=38)
Low/Medium expression + Gleason-score7 (n=172)
Low/Medium expression + Gleason-score8 (n=53)
Low/Medium expression + Gleason-score9 (n=105)
Low/Medium expression + Gleason-score10 (n=3)

Figure S5

A

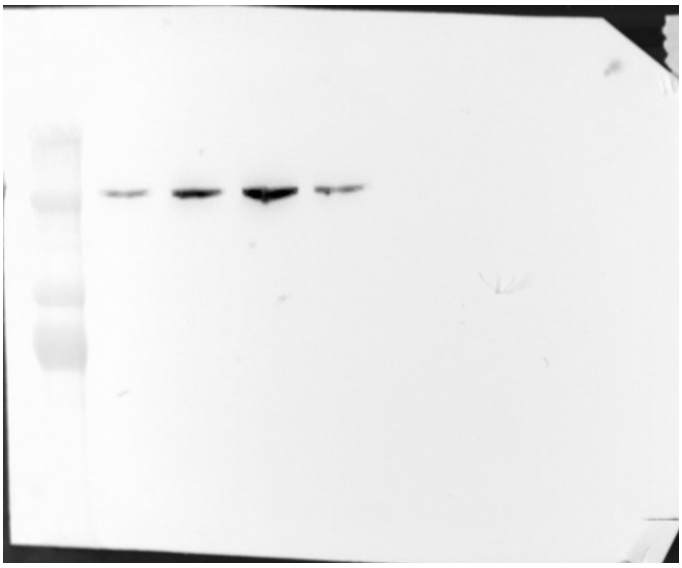

B

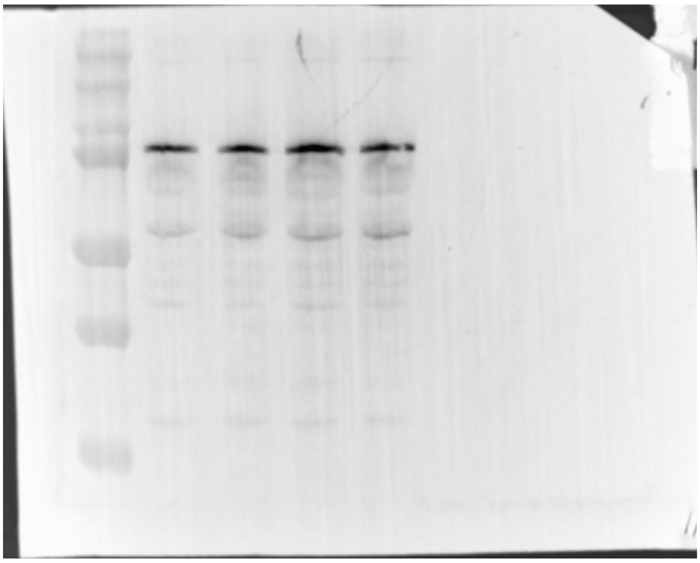

C

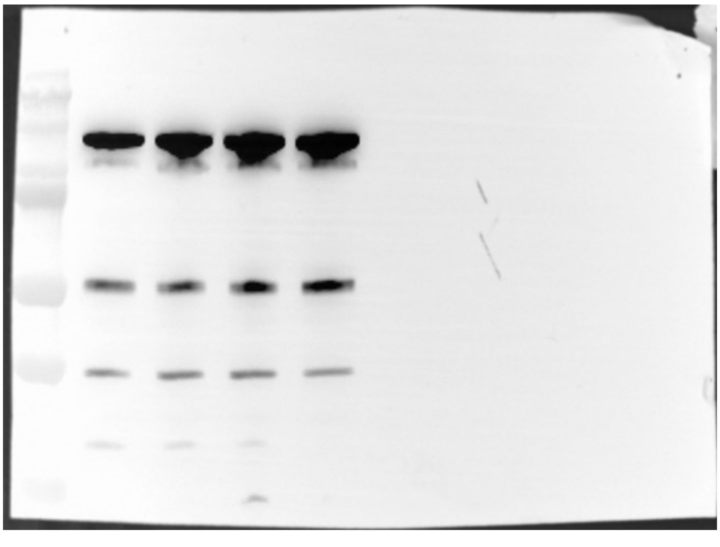

Figure S6

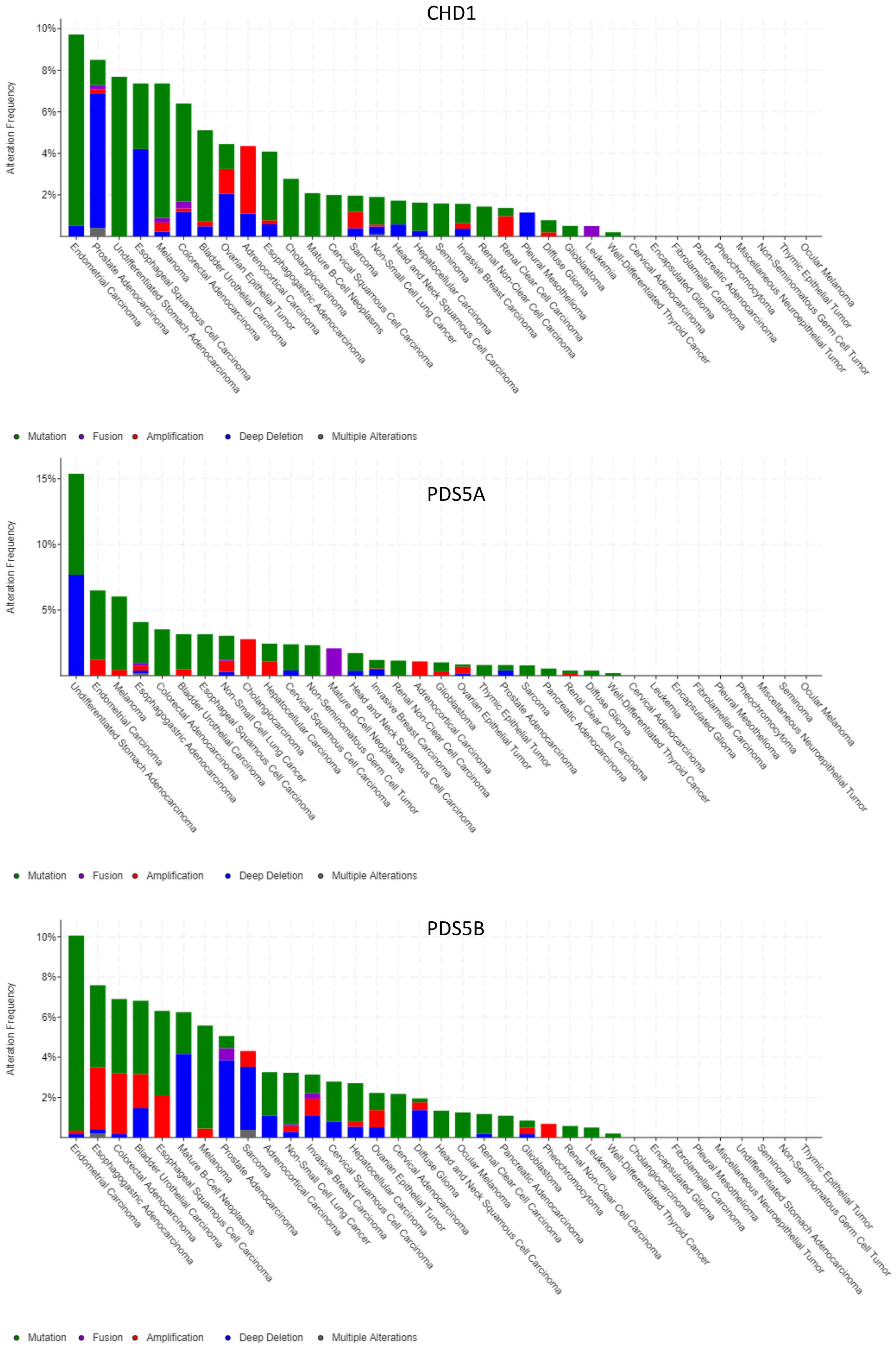
CHD1
Alteration Frequency
10%
8%
6%
4%
2%
Endometrial Carcinoma
Prostate Adenocarcinoma
Undifferentiated Stomach Adenocarcinoma
Esophageal Squamous Cell Carcinoma
Melanoma
Colorectal Adenocarcinoma
Bladder Urothelial Carcinoma
Ovarian Epithelial Tumor
Adrenocortical Carcinoma
Esophagogastric Adenocarcinoma
Cholangiocarcinoma
Mature B-Cell Neoplasms
Cervical Squamous Cell Carcinoma
Sarcoma
Non-Small Cell Lung Cancer
Head and Neck Squamous Cell Carcinoma
Hepatocellular Carcinoma
Seminoma
Invasive Breast Carcinoma
Renal Non-Clear Cell Carcinoma
Renal Clear Cell Carcinoma
Pleural Mesothelioma
Diffuse Glioma
Glioblastoma
Leukemia
Well-Differentiated Thyroid Cancer
Cervical Adenocarcinoma
Encapsulated Glioma
Fibrolamellar Carcinoma
Pancreatic Adenocarcinoma
Pheochromocytoma
Miscellaneous Neuroepithelial Tumor
Non-Seminomatous Germ Cell Tumor
Thymic Epithelial Tumor
Ocular Melanoma
Mutation
Fusion
Amplification
Deep Deletion
Multiple Alterations
PDS5A
Alteration Frequency
15%
10%
5%
Undifferentiated Stomach Adenocarcinoma
Endometrial Carcinoma
Melanoma
Esophagogastric Adenocarcinoma
Colorectal Adenocarcinoma
Bladder Urothelial Carcinoma
Esophageal Squamous Cell Carcinoma
Non-Small Cell Lung Cancer
Cholangiocarcinoma
Hepatocellular Carcinoma
Cervical Squamous Cell Carcinoma
Non-Seminomatous Germ Cell Tumor
Mature B-Cell Neoplasms
Head and Neck Squamous Cell Carcinoma
Invasive Breast Carcinoma
Renal Non-Clear Cell Carcinoma
Adrenocortical Carcinoma
Glioblastoma
Ovarian Epithelial Tumor
Thymic Epithelial Tumor
Prostate Adenocarcinoma
Sarcoma
Pancreatic Adenocarcinoma
Renal Clear Cell Carcinoma
Diffuse Glioma
Well-Differentiated Thyroid Cancer
Cervical Adenocarcinoma
Leukemia
Encapsulated Glioma
Fibrolamellar Carcinoma
Pleural Mesothelioma
Pheochromocytoma
Miscellaneous Neuroepithelial Tumor
Seminoma
Ocular Melanoma
Mutation
Fusion
Amplification
Deep Deletion
Multiple Alterations
PDS5B
Alteration Frequency
10%
8%
6%
4%
2%
Endometrial Carcinoma
Esophagogastric Adenocarcinoma
Colorectal Adenocarcinoma
Bladder Urothelial Carcinoma
Esophageal Squamous Cell Carcinoma
Mature B-Cell Neoplasms
Melanoma
Prostate Adenocarcinoma
Sarcoma
Adrenocortical Carcinoma
Non-Small Cell Lung Cancer
Invasive Breast Carcinoma
Cervical Squamous Cell Carcinoma
Hepatocellular Carcinoma
Ovarian Epithelial Tumor
Cervical Adenocarcinoma
Diffuse Glioma
Head and Neck Squamous Cell Carcinoma
Ocular Melanoma
Renal Clear Cell Carcinoma
Pancreatic Adenocarcinoma
Glioblastoma
Pheochromocytoma
Renal Non-Clear Cell Carcinoma
Leukemia
Well-Differentiated Thyroid Cancer
Cholangiocarcinoma
Encapsulated Glioma
Fibrolamellar Carcinoma
Pleural Mesothelioma
Miscellaneous Neuroepithelial Tumor
Undifferentiated Stomach Adenocarcinoma
Seminoma
Non-Seminomatous Germ Cell Tumor
Thymic Epithelial Tumor
Mutation
Fusion
Amplification
Deep Deletion
Multiple Alterations

Figure S7

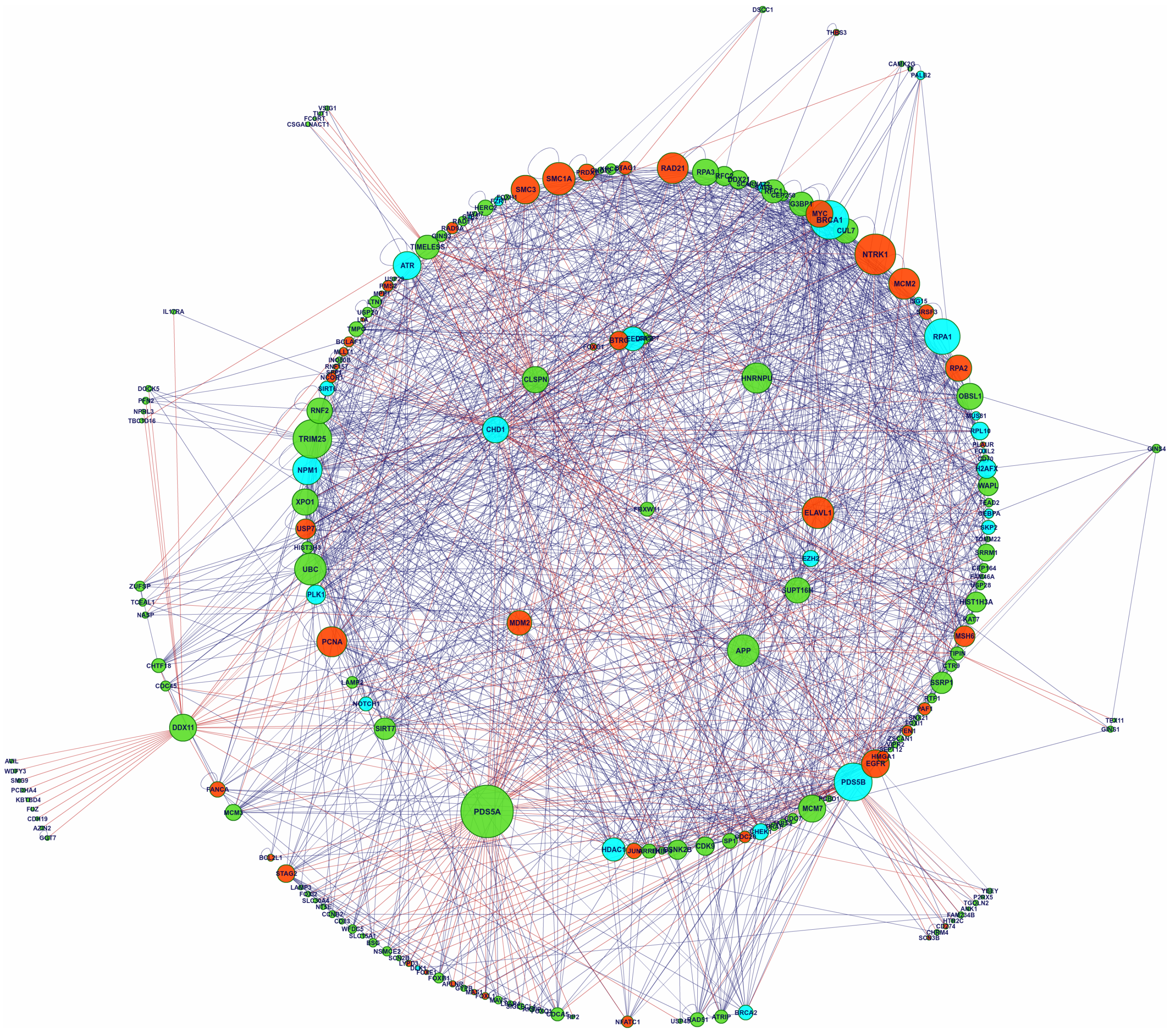

PDS5A
PDS5B
BRCA1
NTRK1
SMC1A
SMC3
RAD21
RPA3
RPA1
RPA2
MCM2
MYC
CUL7
G3BP1
ATR
TIMELESS
CLSPN
HNRNPU
CHD1
RNF2
TRIM25
NPM1
XPO1
USP7
UBC
PLK1
PCNA
MDM2
APP
ELAVL1
SUPT16H
EZH2
FBXW11
OBSL1
WAPL
HIST1H3A
MSH6
SSRP1
EGFR
HMGA1
MCM7
HDAC1
CDK9
SIRT7
NOTCH1
DDX11
FANCA
MCM3
STAG2
CHTF18
CDC45
BRCA2
NFATC1
ATRIP

Supplementary table S1

Strain	Genotype
yIO001	MATa, pep4Δ::g418::TRP1 trp1-1 leu2-3,112 ura3-52 his3-11,15 bar1 GAL+
yIO028	MATa, trp1 ura3 bar1 gal1 PDS5-6MYC
yIO120	MATa, pHIS3-GFPLacI-HIS3: his3-11,15 bar1 trp1-1 leu2-3,112ura3-52, GAL+,SCC2-3V5-G418 LacO-cloNAT::lys4
yIO163	MATa, RAD5+RDN1::ADE2
yIO164	MATa, RAD5+RDN1::ADE2 sir2Δ::TRP
yIO230	MATa, leu2-3,112 ura3-52 his3-11,15 bar1 GAL+, Scc3-6HA, G418
yIO2003	MAT a,pep4Δ::g418::TRP1 trp1-1 leu2-3,112 ura3-52 his3-11,15 bar1 GAL+, chd1Δ::GenMx4
yIO2004	MATa, pep4Δ::g418::TRP1 trp1-1 leu2-3,112 ura3-52 his3-11,15 bar1 GAL+,PDS5-6MYC, CHD1-V5,GenMx4
yIO2005	MATa, pep4Δ::g418::TRP1 trp1-1 leu2-3,112 ura3-52 his3-11,15 bar1 GAL+,CHD1-V5,GenMx4
yAB2010	MATa, pep4Δ::g418::TRP1 trp1-1 leu2-3,112 ura3-52 his3-11,15 bar1 GAL+, chd1Δ::GenMx4,cloNAT lys4::LacO,GFP-LacI-HIS3
yAB2018	MATa, pep4Δ::g418::TRP1 trp1-1 leu2-3,112 ura3-52 his3-11,15 bar1 GAL+, chl1Δ::cloNAT
yAB2019	MATa, pep4Δ::g418::TRP1 trp1-1 leu2-3,112 ura3-52 his3-11,15 bar1 GAL+, chd1Δ::GenMx4, chl1Δ::cloNAT
yAB2021	MATa, pep4Δ::g418::TRP1 trp1-1 leu2-3,112 ura3-52 his3-11,15 bar1 GAL+,PDS5-6MYC, CHL1-V5,GenMx4
yAB2022	MATa, pep4Δ::g418::TRP1 trp1-1 leu2-3,112 ura3-52 his3-11,15 bar1 GAL+,PDS5-6MYC, BRN1-V5,GenMx4
yAB2028	MATa, pHIS3-GFPLacI-HIS3: his3-11,15 bar1 trp1-1 leu2-3,112ura3-52, GAL+, SCC2-3V5-G418 LacO(DK) -cloNAT::lys4, chl1Δ::hygR
yAB2029	MATa, pep4Δ::g418::TRP1 trp1-1 leu2-3,112 ura3-52 his3-11,15 bar1 GAL+,cloNAT lys4::LacO,GFP-LacI-HIS3, chd1Δ::GenMx4, chl1Δ::hygR
yAB2037	MATa, pep4Δ::g418::TRP1 trp1-1 leu2-3,112 ura3-52 his3-11,15 bar1 GAL+, CHL1-V5 GenMx4
yAB2039	MATa RAD5+RDN1::ADE2 chd1Δ::GenMx4
yAB2041	MATa, pep4Δ::g418::TRP1 trp1-1 leu2-3,112 ura3-52 his3-11,15 bar1 GAL+, BRN1-V5 GenMx4
yAB2043	MATa RAD5+RDN1::ADE2 chd1Δ::GenMx4 chl1Δ::cloNAT
yAB2044	MATa RAD5+RDN1::ADE2 chl1Δ::cloNAT
yAB2045	MATa, leu2-3,112 ura3-52 his3-11,15 bar1 GAL+, Scc3-6HA,G418, chl1Δ::cloNAT
yAB2046	MATa leu2-3,112 ura3-52 his3-11,15 bar1 GAL+, Scc3-6HA,G418, chd1Δ::GenMx4
yAB2048	MATa leu2-3,112 ura3-52 his3-11,15 bar1 GAL+, Scc3-6HA,G418, chd1Δ::GenMx4, chl1Δ::cloNAT
yAM-895	MATa, leu2-3,112 ura3-52 his3-11,15 bar1 GAL+ cloNAT lys4::LacO GFP-LacI-HIS3 tof1Δ::HPH
yAM903	MATa, leu2-3,112 ura3-52 his3-11,15 bar1 GAL+ cloNAT lys4::LacO GFP-LacI-HIS3 tof1Δ::HPH chd1 Δ::G418

Supplementary table S2

Locus	Location	Forward/ Reverse	Primer (5' -> 3')
Chromosome III arm	99327 kb	Forward	AGC GGA TCA ATC CAC AAA GC
		Reverse	TGC TGT AGT CAC CTC AGC AAG
	99690 kb	Forward	AAA GGT GCC CCA AGA AAA GG
		Reverse	AGC ACT TTA CTC GCT TGT GG
	100235 kb	Forward	ATG CCA AGG CGG AAA GAA TG
		Reverse	TGG GGG CTT CTC GAT TTT TG
	100925 kb	Forward	ATG AGA AAG AGG GGT TCC TTC G
		Reverse	GGC GTC AAT GCT TTA GTT CTC C
	101809 kb	Forward	ACT TTG GTT TTC CGG TGT GC
		Reverse	CCA GCG ATG AGA TGC GAA AAG
	102140 kb	Forward	ATG GTT CGG TTG GTG CTT AG
		Reverse	ACG CGG AAT TGA AAC CAC AG
	101956 kb	Forward	TCG CTT TTC GCA TCT CAT CG
		Reverse	AGC GGG CGG GTT ATA AAT AAC
Chromosome IV centromere	445412-445505	Forward	GGTTGGGATCTAGGGATTAC
		Reverse	TGATTGATTCACCTAGCCTT
	449646-449728	Forward	ACACGAGCCAGAAATAGTAAC
		Reverse	TGATTATAAGCATGTGACCTTT
	451211-451408	Forward	GGAATACCGAGACCGTTAG
		Reverse	ACAGCCCCCATTCTTG
	452348-452462	Forward	CTTGGGTCTGTTGGGG
		Reverse	ATACAAGCCAAGGACCG
Chromosome XII rDNA (cohesin)	NTS2 (1)	Forward	CGTTCATAGCGACATTGCTT
		Reverse	GGGTGAACAATCCAACGCTT
	3' 35S (2)	Forward	TGTTAGTGCAGGAAAGCGGG
		Reverse	CTACACCCTCGTTTAGTTGC
	5' 35S (3)	Forward	GTATGTGGGACAGAATGTCG
		Reverse	GACTTACGTTTGCTACTCTC
	NTS1 (4)	Forward	GCTTGCGTTGATTACGTCCC
		Reverse	CACTAAGCCATTCAATCGGT
Chromosome XII rDNA (condensin)	NTS2 (I)	Forward	ATATGAGGGCAGGGTCCAGACATGTT
		Reverse	ACCTGTCACCTTGAAACTACCTCTGC
	3' 35S(II)	Forward	TTTGTATGTTCCCGCGCGTTTCCGTA
		Reverse	CACTTGTACTCCATGACTAAACCCCC
	5' 35S(III)	Forward	GAGGGTGTAGAATAAGTGGGAGCTTC
		Reverse	AGGATCGATAGGCCACACTTTCATGG
	NTS1 pre rfb (IV)	Forward	AGCCTACTCGAATTCGTTTCC
		Reverse	ATAGTGAGGAACTGGGTTACC
	Rfb (V)	Forward	GTGAAAGGATTTGCCCGGACAGTTTG
		Reverse	CCCACTGTTCACTGTTCACTGTTCAC
	NTS1 post rfb (VI)	Forward	AGGCAGCGTAAAAGGATGAGGCTACT
		Reverse	TTCCTTCCCGCTTTCCTGCACTAACA
